# PARP抑制剂在小细胞肺癌的研究进展

**DOI:** 10.3779/j.issn.1009-3419.2020.102.19

**Published:** 2020-09-20

**Authors:** 爽 张, 菁菁 柳, 长良 杨, 良 张, 双 李, 昊 暴, 颖 程

**Affiliations:** 130012 长春，吉林省肿瘤医院胸部肿瘤内科 Department of Thoracic Oncology, Jilin Provincial Cancer Hospital, Changchun 130012, China

**Keywords:** 肺肿瘤, 聚腺苷二磷酸核糖多聚酶抑制剂, Lung neoplasms, PARP inhibitor

## Abstract

以早期转移，容易复发耐药，预后差为特征的小细胞肺癌（small cell lung cancer, SCLC）的治疗依然面临困境。在免疫治疗为SCLC带来新的选择后，研究者也热切的希望SCLC能够在靶向治疗领域实现突破。SCLC基因组不稳定性和对细胞毒性化疗的敏感性，使得靶向脱氧核糖核酸（deoxyribonucleic acid, DNA）修复相关途径的药物聚腺苷二磷酸核糖多聚酶（poly ADP-ribose polymerase, PARP）抑制剂成为SCLC靶向治疗研究的热点。目前PARP抑制剂在SCLC的研究既有单药治疗，也有联合其他药物的治疗策略，既包括复发SCLC的治疗和SCLC的一线诱导治疗，也包括诱导治疗后的维持治疗，这些研究也分别进行了的疗效预测标志物的探索，尽管目前PARP抑制剂在SCLC的研究结果有限，发现的疗效预测标志物也不一致，但我们也看到PARP抑制剂可能是SCLC精准治疗的突破口。

## Abstract (en)

Small cell lung cancer (SCLC), characterized by early metastasis, relapse, relapse and resistance and poor prognosis, still faces difficulties in treatment. Recently, Immunotherapy is a novel treatment for SCLC, researchers are also eager to achieve a breakthrough in targeted treatment of SCLC. Genomic instability of SCLC and sensitivity to cytotoxic chemotherapy, therefore, poly ADP-ribose polymerase (PARP) inhibitors targeting DNA repair related pathways have become a hotspot in the research of SCLC targeted therapy. Studies on PARP inhibitors in SCLC have been conducted in combination with other therapeutic strategies, including the treatment of recurrent SCLC and first-line treatment, as well as maintenance treatment after induction. These studies also explored the predictive markers of PARP inhibitors in SCLC. Although the current results of PARP inhibitors in SCLC are limited, and the predictive markers are also inconsistent, we also see that PARP inhibitors could be a breakthroughfor precision medicine of SCLC.

## 1 前言

小细胞肺癌（small cell lung cancer, SCLC）是最具侵袭性的肺癌亚型，肿瘤倍增时间短，早期出现转移，5年的生存率不足7%^[1]^。近40年来细胞毒性化疗一直是SCLC治疗的基石。虽然免疫治疗为SCLC带来新的治疗选择，但在靶向治疗领域依然进展缓慢。SCLC基因组不稳定，普遍存在TP53和RB1失活，这使得肿瘤细胞的存活更加依赖DNA损伤应答和细胞周期阻滞^[2]^，因此针对这些途径相应靶点的治疗在SCLC充满应用的前景。

聚腺苷二磷酸核糖多聚酶（poly ADP-ribose polymeras, PARP）是一种脱氧核糖核酸（deoxyribonucleic acid, DNA）修复蛋白，其功能是检测和标记DNA单链损伤，并连接到DNA损伤位点，合成腺苷二磷酸（adenosine diphosphate, ADP）核糖链，募集大量的脚架蛋白和DNA修复酶去修复单链的损伤^[3]^。当PARP功能受损时，发生持续的单链DNA损伤，单链DNA损伤不断累积而发生双链DNA断裂，双链DNA断裂通过同源重组进行修复，如果此时同源重组受损，断裂的双链DNA将不能进行同源重组修复。乳腺癌易感基因（breast cancer susceptibility gene, *BRCA*）*1/2*是参与同源重组修复的重要基因，*BRCA1/2*突变的肿瘤，如果同时存在PARP功能受损就会发生协同致死作用^[4, 5]^。在蛋白组和转录组的整合分析中，发现SCLC中PARP1蛋白表达上调，而且高于其他类型的肺癌^[6]^。虽然SCLC中*BRCA1/2*突变非常罕见（≤3%）^[2, 7]^，但体外研究^[6]^发现SCLC细胞系对PARP抑制剂仍然敏感，提示B*RCA1/2*突变之外的DNA修复通路的异常也影响肿瘤对细胞对PARP抑制剂的敏感性^[8, 9]^。目前PARP抑制剂在SCLC的研究仍然处在探索阶段，研究涵盖一线、二线和维持治疗，以联合治疗为主，多为Ⅰ期/Ⅱ期研究，寻找能够使SCLC从PARP抑制剂治疗中获益的潜在的标志物是研究的热点领域。

## 2 PARP抑制剂单药在SCLC中的研究

PARP抑制剂单药在SCLC中进行了初步的尝试。临床前研究发现，在35种不同类型的肺癌细胞系中，SCLC细胞系对PARP抑制剂最敏感^[6]^，提示PARP抑制剂单药在SCLC中值得进行探索。他拉唑帕尼（Talazoparib）是一种强效的PARP1/2抑制剂，在一项他拉唑帕尼治疗实体瘤的剂量爬坡-扩增两阶段Ⅰ期研究的剂量扩增阶段纳入了SCLC共23例，患者既往治疗的中位周期数为1，患者接受他拉唑帕尼1.0 mg/d治疗，其中有2例患者获得部分缓解（partial response, PR），这2例患者应答持续时间分别为12.0周和15.3周，均为敏感复发患者；有4例患者疾病稳定（stable disease, SD），持续至少16周，临床获益率为26%，中位无进展生存期（progression-free survival, PFS）为11.1周^[10]^。奥拉帕尼（Olaparib）是第一个获得美国食品药品管理局（Food and Drug Administration, FDA）批准上市的PARP抑制剂，在一项进行标志物筛选的SCLC伞状研究中（SUKSES）^[11]^，B队列（NCT03009682）为存在同源重组缺陷的复发SCLC患者接受奥拉帕尼单药治疗，目前纳入9例患者，8例患者评价了疗效，没有完全缓解（complete response, CR）和PR的患者，2例患者最佳疗效为SD，6例患者疾病进展（progressive disease, PD），这项研究仍在进行中。PARP抑制剂单药在复发SCLC中有一定的抗肿瘤活性，在肿瘤负荷更低的经过诱导化疗后疾病没有的进展的SCLC患者中，应用PARP抑制剂进行维持治疗是否能够延缓肿瘤复发也是探索的方向之一。目前一项尼拉帕尼（Niraparib）对比安慰剂在广泛期小细胞肺癌（extensive-stage small cell lung cancer, ES-SCLC）进行维持治疗的Ⅲ期研究正在进行（NCT03516084），期待这项研究能够回答PARP抑制剂在SCLC中延缓疾病进展的意义。

## 3 PARP抑制剂联合化疗在SCLC中的研究

尽管临床前研究和早期的临床研究中看到PARP抑制剂治疗SCLC的前景，然而PARP抑制剂单药的作用非常有限，理论上与增加DNA单链损伤的药物联合应用将会进一步增加疗效，发挥协同作用。在多种实体瘤中的研究中发现PARP抑制剂与DNA损伤剂替莫唑胺联合发挥协同作用^[12]^。SCLC动物模型中他拉唑帕尼和替莫唑胺在1:700到1:700, 000的比例联合治疗时显著超过单药的活性^[12]^。

PARP抑制剂与PARP1和/或PARP2的烟酰胺腺嘌呤二核苷酸^+^（nicotinamide adenine dinucleotide^+^, NAD^+^）结合，阻止PARP与DNA的分离，导致无法进行后续的DNA修复，称为PARP捕获（trapping）。PARP抑制剂按照PARP捕获能力排序为talazoparib≫niraparib > olaparib=rucaparib≫veliparib^[13]^。研究^[14]^还发现，SCLC细胞系中PAPR抑制剂的活性与PARP捕获的能力相关，提示在SCLC中具有更高PARP捕获的抑制剂具有更高的活性。研究者采用生物信息分析发现在SCLC细胞系中，*SLFN11*是与PARP抑制剂治疗敏感性相关性最高的基因之一，在转录水平和蛋白水平均发现SLFN11高表达的SCLC对PARP抑制剂治疗更敏感，而敲除SLFN11后，SCLC细胞则对PARP抑制剂耐药^[14]^。人的*Slfn11*基因编码的蛋白与解旋酶结构相似的，复制蛋白A（replication protein A, RPA）是真核生物单链DNA连接蛋白，是DNA复制，重组和DNA修复等多种DNA代谢途径中所必须的蛋白，SLFN11以RPA依赖的模式连接到DNA损伤的部位，影响RPA-DNA复合物稳定性，继而抑制同源重组修复^[15]^，PARP抑制剂在SLFN11异常表达的情况下，也会发生协同致死作用，提示SLFN11可能是PAPR抑制剂治疗SCLC的疗效预测标志物。

一项Ⅱ期研究比较了替莫唑胺联合维利帕尼（Veliparib）或者安慰剂治疗复发SCLC的疗效和安全性，研究纳入104例至少接受一个方案治疗的复发SCLC患者^[16]^，研究的主要终点是4个月的PFS。在非选择人群中，无论是4个月的PFS率，还是中位PFS和总生存（overall survival, OS），维利帕尼联合替莫唑胺与安慰剂联合替莫唑胺都没有显著的差异。进一步的探索性分析发现，在维利帕尼联合替莫唑胺组，与SLFN11免疫组化阴性表达的患者相比，阳性表达的患者有更长的中位PFS（5.7个月*vs* 3.6个月，*P*=0.009）和OS（12.2个月*vs* 7.5个月，*P*=0.014），而在替莫唑胺联合安慰剂组，SLFN11免疫组化表达情况不影响患者的PFS和OS。这项研究也是首次在临床研究中发现SLFN11是预测SCLC患者能够从PARP抑制剂治疗中获益的标志物。

而一项奥拉帕尼联合替莫唑胺治疗复发SCLC的Ⅰ期/Ⅱ期研究^[17]^在标志物探索方面却有不同的发现。这项研究纳入了50例患者，确认的客观缓解率（objective response rate, ORR）为41.7%（20/48），中位的PFS和OS分别为4.2个月和8.5个月，从初步的结果中看到奥拉帕尼联合替莫唑胺具有非常良好的疗效。这项研究中也建立了患者来源的移植瘤模型（patient-derived xenografts, PDX）探索能够预测奥拉帕尼联合替莫唑胺治疗SCLC的分子特征。通过对PDX模型RNA的富集分析发现24个炎症相关基因富集在对奥拉帕尼联合替莫唑胺治疗敏感的患者中，研究者选择其中4基因[3个干扰素诱导表达的基因：*CEACAM1*、*TNFSF10*、*OAS1*和转化生长因子β（transforming growth factor β, TGFβ）诱导表达的基因*TGIF1*]进行验证，在奥拉帕尼联合替莫唑胺治疗敏感的PDX中CEACAM1、TNFSF10和TGIF1在表达水平显著升高，OAS1可以作为补充的标志物，与其他基因联合检测增加特异性，而MYC靶基因（*EIF4A1*）与奥拉帕尼联合替莫唑胺治疗耐药相关。

从上面的两项研究中看到，SCLC不同的研究中发现的预测PARP抑制剂疗效的标志物不同，这可能与不同的研究研究设计、选择的动物模型和治疗策略等因素的差异有关。

除了在复发SCLC中进行了探索，PARP抑制剂联合化疗也在ES-SCLC一线治疗进行了尝试。一项Ⅱ期研究探索了化疗联合维利帕尼或者安慰剂治疗一线ED-SCLC的疗效和安全性，主要终点为PFS^[18]^，研究按照性别，乳酸脱氢酶（lactate dehydrogenase, LDH）水平进行分层。该研究共纳入了128例患者，维利帕尼联合化疗与安慰剂联合化疗在中位PFS和OS都没有显著的差异，亚组分析发现，男性且LDH高于正常的患者接受维利帕尼联合化疗可以获得PFS的改善。目前对于男性且LDH升高的患者能够从PARP抑制剂联合标准化疗中获益也缺少生物学上的合理解释，可能是由于这个亚组与其他亚组相比有最大的样本量，而这个样本量包含了足够比例的具有某种分子异常的能够从PARP抑制剂联合化疗中获益的患者。从目前的PARP抑制剂联合化疗的研究中可以看到，非选择人群中在化疗的基础上增加PARP抑制剂并没有显著提高疗效，PARP抑制剂联合化疗治疗SCLC患者只有在选择人群中进行才有意义，现有的数据中只有SLFN11免疫组化表达是在动物模型中发现，又经过临床研究小样本亚组患者证实的，但是SLFN11的表达受既往化疗的影响，而炎症相关基因异常表达与PARP抑制剂疗效的相关性仅在动物模型中发现并没有在临床研究中验证，可见SCLC的PARP抑制剂疗效预测标志物还需要进行大量的研究去明晰。

另外也有几项PARP抑制剂联合化疗治疗SCLC的研究正在进行。维利帕尼联合拓扑替康治疗复发SCLC的Ⅰ期研究（NCT03227016），CRLX101（纳米喜树碱）联合奥拉帕尼治疗复发SCLC的Ⅰ期/Ⅱ期研究（NCT02769962），他拉唑帕尼联合低剂量的替莫唑胺治疗复发ES-SCLC的Ⅱ期研究（NCT03672773），以及尼拉帕尼联合替莫唑胺联合作为ES-SCLC一线治疗后CR/PR患者维持治疗的Ⅰ期/Ⅱ期研究（NCT03830918）等，期待这些研究结果能够PARP抑制剂联合化疗治疗SCLC提供更多的证据支持。

## 4 PARP抑制剂联合免疫治疗在SCLC中的研究

最近针对程序性细胞死亡受体-1/程序性细胞死亡配体-1（programmed cell death-1/programmed cell death-ligand 1, PD-1/PD-L1）免疫检查点的单克隆抗体在多种实体瘤中获得突破，也为SCLC的治疗带来希望。PD-L1抑制剂阿特珠单抗（Atezolizumab）和德瓦鲁单抗（Durvalumab）联合标准化疗带来ES-SCLC生存的获益^[19, 20]^，建立ES-SCLC一线治疗新的标准，然而与标准化疗相比，2个月和2.7个月生存的延长并未使SCLC的预后得到彻底的改观。纳武单抗（Nivolumab）和帕博利珠单抗（pembrolizumab）虽然改变了SCLC三线及后线无标准治疗的窘境^[21, 22]^，然而不足20%的ORR和有限的PFS更是差强人意。联合治疗是改善免疫治疗在SCLC疗效的策略之一。DNA损伤与免疫应答相关，提示PARP抑制剂与免疫治疗能够发挥协同作用，是有希望的联合策略。

存在*BRCA*突变或者其他同源重组缺陷的肿瘤有较高的新抗原负荷和肿瘤突变负荷，肿瘤组织中CD8/CD4增加，浸润的免疫细胞增多，PD-L1表达上调，对于这样的肿瘤PD-1/PD-L1抑制剂能够增加PARP抑制剂的抗肿瘤活性^[23]^。另外PARP抑制剂使内源性DNA损伤累积，触发细胞质DNA感受器环GMP-AMP合酶（cyclic GMP-AMP synthase, cGAS），促进第二信使cGAMP释放，活化STING信号，使干扰素调节因子（interferon regulating factor 3, IRF3）磷酸化，并进入细胞核，诱导炎症相关基因转录表达[β-干扰素（interferon-β, IFN-β）、白细胞介素-5（interleukin 5, IL-5）、白细胞介素-10（interleukin 10, IL-10）表达]，使T细胞募集、活化，促进免疫细胞浸润肿瘤组织，肿瘤细胞PD-L1表达上调^[24-26]^，诱导抗肿瘤免疫，因此PARP抑制剂与PD-1/PD-L1抑制剂相互作用，而发挥协同抗肿瘤作用。

临床前研究发现，DNA损伤应答（DNA damage response, DDR）抑制剂（例如PARP抑制剂和检查点激酶1（checkpoint kinase 1, CHK1）抑制剂使SCLC细胞系PD-L1表达增加3倍以上，而且呈时间依赖性，在SCLC鼠模型中，免疫联合DDR抑制剂使CD3^+^ T，CD8^+^ T显著增加，CD4^+^_helper_T、Treg、耗竭型T细胞明显减少，能够显著抑制肿瘤生长，延长鼠SCLC模型的生存时间^[24]^。深入的机制探索发现SCLC中内源性DNA损伤是由DDR进行修复，PARP抑制剂导致细胞质DNA释放，活化cGAS-STING信号，显著增强PD-1/PD-L1抑制剂的疗效，这项临床研究也探索了PARP抑制剂联合免疫治疗复发SCLC的疗效。一项单臂、开放标签奥拉帕尼联合德瓦鲁单抗治疗复发SCLC的Ⅱ期研究中^[27]^，纳入20例复发SCLC患者，其中一半的患者接受2个或2个以上方案治疗，60%的患者为耐药复发的SCLC，在19例可评价疗效的患者中，1例为经确认的CR，1例为PR，4例患者SD，ORR为10.5%，中位的PFS和OS分别为1.8个月和4.1个月。这项研究也进一步探索了PARP抑制剂联合免疫治疗获益的潜在标志物，19例患者提供了治疗前的标本，18例有充足肿瘤组织评估了PD-L1表达和淋巴细胞浸润情况，发现PD-L1表达与联合治疗无显著相关性。研究者根据患者肿瘤组织PD-L1表达和淋巴细胞浸润情况将SCLC分为不同的免疫表型，同时评估不同免疫表型与奥拉帕尼联合德瓦鲁单抗治疗疗效的相关性，发现对治疗有应答的患者为有成簇浸润的T细胞广泛的分布于肿瘤内部，同时肿瘤细胞和浸润的淋巴细胞有PD-L1表达增加的炎症型，提示免疫表型为炎症型的患者可能是奥拉帕尼联合德瓦鲁单抗治疗潜在获益的人群。对获得CR的患者进行了基因分析发现该患者存在*BRCA1*突变，可能与DNA损伤修复缺陷使SCLC对PARP抑制剂敏感，而免疫治疗增加应答的深度，延迟了应答持续时间，使患者获得了CR的疗效，由于这项研究中预测标志物的分析受到样本量、肿瘤标本取材时间、肿瘤的异质性等诸多的因素的影响尼拉帕尼联合纳武单抗治疗复发SCLC的Ⅱ期研究（NCT03958045）也正在进行，未来还需进行更多的探索。

## 5 总结

PARP抑制剂让SCLC的靶向治疗燃起了希望，基础研究为PARP抑制剂在SCLC治疗策略的选择提供线索，目前PARP抑制剂在SCLC的临床研究陆续开展，除了在复发SCLC中PARP抑制剂单药治疗外，更多的研究为PARP抑制剂联合其他药物治疗SCLC。虽然目前的研究多为小样本的Ⅰ期/Ⅱ期研究，数据非常有限，标志物的探索也没有获得一致的结果，但是我们看到SLFN11表达，炎症相关基因在PARP抑制剂治疗SCLC中预测疗效的前景。一直以来SCLC是作为不加区分的整体进行治疗的。随着最近研究的进展，根据4个关键的转录因子表达的差异将SCLC初步分为4种亚型^[28]^，其中P亚型（POU 2F3高表达）潜在的对PARP抑制剂治疗更敏感，随着更多的研究开展和结果的公布，PARP抑制剂将成为SCLC实现精准治疗的突破口。
